# Multi-omics integration reveals BPGM downregulation and potential plasma metabolite biomarkers for childhood asthma

**DOI:** 10.3389/fped.2026.1794811

**Published:** 2026-05-07

**Authors:** Junlin Zhao, Zhiyuan Wang, Yanan Wang, Qianqian Dai, Menghua Li, Aliya Maimaitiniyazi, Zhenzhen Guo, Liang Ru

**Affiliations:** Department of Pediatrics, The First Affiliated Hospital of Xinjiang Medical University, Urumqi, Xinjiang, China

**Keywords:** BPGM, childhood asthma, diagnostic biomarkers, glycine-Serine-Threonine metabolism, metabolomics, transcriptomics

## Abstract

**Objective:**

This study aimed to identify potential diagnostic biomarkers and candidate therapeutic targets through multi-omics integration of peripheral blood transcriptomics and metabolomics in children with asthma. We specifically investigated the association between bisphosphoglycerate mutase (BPGM) downregulation and metabolic alterations in the glycine-serine-threonine pathway, seeking to explore potential mechanisms underlying asthma-related metabolic reprogramming.

**Methods:**

In this exploratory multi-omics study, we integrated public transcriptomic data (GSE35571, *n* = 124 samples: 60 asthma, 64 controls) with in-house untargeted metabolomics (*n* = 30 samples: 15 asthma, 15 controls) from treatment-naive, normal-weight children aged 6–14 years. The transcriptomic cohort was derived from a US population (Detroit, Michigan) while the metabolomic cohort was from Xinjiang, China. While this cross-population design precludes direct gene–metabolite correlation at the individual level, pathway-level convergence across ethnically and geographically distinct cohorts may suggest conserved disease-related biological processes, although the pathway intersection should be interpreted as indirect concordance supporting hypothesis generation rather than establishing mechanistic linkage.

**Results:**

Transcriptomics identified 15 differentially expressed genes (*p* < 0.05, |log_2_FC| > 0.25), including significant BPGM downregulation (log_2_FC = −0.2731, *p* = 0.0422). Metabolomics revealed 516 differential metabolites [*p* < 0.05, variable importance in projection (VIP) > 1]. Kyoto Encyclopedia of Genes and Genomes (KEGG) pathway intersection identified glycine-serine-threonine metabolism (hsa00260) as the core shared pathway, enriched with BPGM and three upregulated metabolites: L-tryptophan, 5-aminolevulinic acid, and L-aspartate semialdehyde. These metabolites demonstrated good diagnostic performance with area under the curve (AUC) values of 0.818, 0.844, and 0.818, respectively. 5-Aminolevulinic acid showed optimal diagnostic accuracy with 80% sensitivity and 80% specificity. Spearman correlation analysis revealed that 5-aminolevulinic acid was significantly positively correlated with both serum IgE (*r* = 0.469, *p* = 0.009) and eosinophil counts (*r* = 0.506, *p* = 0.004), while no significant correlations were observed with pulmonary function parameters.

**Conclusions:**

This preliminary multi-omics integration study identified concurrent BPGM downregulation and altered glycine-serine-threonine metabolism in childhood asthma. While these findings suggest a potential association between BPGM expression changes and metabolic alterations in this pathway, the proposed mechanistic link remains hypothetical and requires direct experimental validation.

## 1 Introduction

Childhood asthma is the most common chronic respiratory disease in pediatric populations, with a global prevalence of approximately 10.2% (1). This disease exhibits remarkable heterogeneity due to complex interactions among genetic, immunological, environmental, and metabolic factors (2). Although inhaled corticosteroid-based therapies have been widely adopted, some patients experience inadequate symptom control and progress to severe or refractory asthma (3). Therefore, there is an urgent need to elucidate the molecular mechanisms underlying childhood asthma heterogeneity. This will enable the identification of precise endotypes and the development of individualized therapeutic strategies accordingly. The heterogeneity of childhood asthma necessitates precision medicine approaches that can identify distinct molecular endotypes and guide individualized treatment strategies (2). In pediatric populations, this challenge is compounded by age-specific physiological characteristics, developmental changes in immune function, and practical limitations of conventional diagnostic methods. Multi-omics integration offers a powerful approach to dissect this complexity by connecting genetic predisposition, transcriptional regulation, and metabolic phenotypes, thereby revealing actionable therapeutic targets and biomarkers tailored for children.

In recent years, multi-omics approaches, including genomics, transcriptomics, epigenomics, and metabolomics, have been extensively applied to childhood asthma research, substantially expanding our understanding of disease heterogeneity (4, 5). However, most existing studies have predominantly used single-omics platforms, which fail to capture the complete molecular regulatory cascade from gene expression to metabolic phenotypes (6). Multi-omics integration strategies enable the identification of core pathways and key regulatory nodes at different biological levels, yet their application in childhood asthma is still nascent, with few studies combining transcriptomic and metabolomic analyses, in particular (7, 8). Metabolomics research has provided important insights into metabolic dysregulation in asthma. Previous studies have identified aberrations in multiple metabolic pathways in children with severe asthma and acute exacerbations. Among these pathways, the glycine, serine, and threonine metabolism pathway (hsa00260) has attracted considerable attention due to its close association with oxidative stress, one-carbon metabolism, and glutathione synthesis (9, 10) Fitzpatrick et al. first reported disruption of this pathway in children with severe asthma and its correlation with oxidative stress (11); Cottrill et al. further validated the central role of this pathway in acute exacerbations (12). However, these studies focused mainly on describing metabolite changes, lacking exploration of upstream transcriptional regulatory mechanisms. As a result, they have not elucidated the molecular drivers, such as transcription factors or enzymes, underlying these metabolic abnormalities.

Bisphosphoglycerate mutase (BPGM) is a key enzyme in the erythrocyte glycolytic shunt pathway that regulates hemoglobin-oxygen affinity through the synthesis of 2,3-BPG (13). Recent studies have revealed BPGM expression in tissues including kidney, placenta, and astrocytes, where it participates in the regulation of glucose metabolism, oxidative stress responses, and inflammatory reactions (14, 15). This broader tissue distribution prompted Kulow et al. (16) in 2024 to investigate its function further; they reported that a tubule-specific knockout of BPGM resulted in acute kidney injury, suggesting that its role in maintaining tissue metabolic homeostasis extends beyond erythrocytes. These findings position BPGM as a particularly relevant candidate for asthma research through several mechanistic considerations. In the context of asthma pathogenesis, BPGM-mediated regulation of oxygen delivery becomes especially pertinent during acute exacerbations when tissue oxygenation may be compromised. Moreover, its demonstrated involvement in cellular stress responses across multiple tissue types suggests potential roles in airway inflammation and metabolic adaptation. Critically, glycolytic metabolism and amino acid biosynthesis are metabolically interconnected through shared intermediates (17), positioning BPGM as a potential upstream regulator of the glycine-serine-threonine pathway observed to be dysregulated in our study. Despite these compelling connections, the role of BPGM in asthma and its specific association with amino acid metabolic pathways have not been previously investigated, providing strong rationale for the present multi-omics exploration.

Current multi-omics studies in childhood asthma lack systematic integration at the transcription-metabolism interface. Previous metabolomics investigations have predominantly focused on severe asthma or acute exacerbations, with insufficient understanding of early metabolic signatures in normal-weight, treatment-naive patients. Furthermore, the upstream transcriptional regulatory mechanisms underlying disruption of the glycine-serine-threonine metabolism pathway remain unclear, and the role of BPGM in asthma has not been elucidated. With these considerations in mind, the present study integrated a public transcriptomic dataset (GSE35571) with an in-house metabolomics cohort. Through Kyoto Encyclopedia of Genes and Genomes (KEGG) pathway intersection analysis, we identified a putative molecular link from gene expression alterations to metabolite changes. Specifically, we aimed to: First, elucidate the molecular mechanisms underlying childhood asthma through integration of transcriptomic and metabolomic data, Secondly, investigate the role of BPGM and its association with amino acid metabolic pathways in asthma pathogenesis. Finally, identify clinically actionable biomarkers that could facilitate early diagnosis and disease monitoring in pediatric populations. This work provides novel insights into systemic metabolic imbalances associated with childhood asthma and supports the development of precision medicine strategies based on metabolic phenotypes.

## 2 Materials and methods

### 2.1 Metabolomics study subjects

Metabolomics data were collected from normal-weight asthmatic children aged 6–14 years (asthma group, *n* = 15) who presented to the Pediatric Center of the First Affiliated Hospital of Xinjiang Medical University between June 2025 and September 2025, along with age-matched healthy children (control group, *n* = 15). All subjects were gender-matched with a male-to-female ratio of 7:8. Sample size was determined based on pilot metabolomics studies showing adequate statistical power for detecting differential metabolites with effect sizes >1.5.

### 2.2 Inclusion and exclusion criteria

Inclusion criteria for the asthma group: (1) age range 6–14 years; (2) body weight within the normal range according to the “Body mass index (BMI) growth curves for Chinese children and adolescents aged 0 to 18 years” (18); (3) patients who fulfill the Global Initiative for Asthma (GINA) 2025 diagnostic criteria for childhood asthma (19); (4) treatment-naïve patients at initial diagnosis who had not received standardized asthma therapy. Exclusion criteria: (1) comorbid chronic lung diseases; (2) congenital metabolic disorders; (3) congenital heart disease; (4) a definitive diagnosis of epilepsy or intellectual disability.

Sample collection and processing: Venous blood (3–5 mL) was collected from all subjects in the early morning after fasting and collected into EDTA tubes. After centrifugation at 1,000 × g for 10 min at 4 °C to separate plasma, the plasma was aliquoted and stored at −80 °C until analysis.

### 2.3 Transcriptomic differential expression analysis

Transcriptomic data were obtained from the National Center for Biotechnology Information (NCBI) Gene Expression Omnibus (GEO) database (GSE35571). These data originated from the Mechanistic Indicators of Childhood Asthma (MICA) study, funded by the U.S. Environmental Protection Agency, which aimed to explore the relationships among environmental exposures, biomarkers, and childhood asthma. The dataset comprised 124 peripheral blood samples from children aged 9–13 years, from Detroit, Michigan, USA, including 60 asthmatic cases and 64 healthy controls. Peripheral blood samples were collected from children in the Detroit area and subjected to genome-wide expression profiling, using the Affymetrix Human Genome U133 Plus 2.0 array. In the present study, we conducted a secondary analysis of these publicly available data to explore transcriptomic characteristics in normal-weight asthmatic children.

We performed differential expression analysis of the GSE35571 dataset using the limma package in R software (version 4.5.2). The raw expression matrix was first subjected to probe annotation and gene symbol mapping. Unannotated probes were removed, yielding 21,755 genes for subsequent analysis. Following this preprocessing, expression data were fitted to the limma linear model, with standard errors of the estimated coefficients adjusted using empirical Bayes methods. Differentially expressed genes (DEGs) were identified using the criteria of *p* < 0.05, and |log_2_ fold change (log_2_FC)| > 0.25. We adopted a relatively modest fold-change threshold (|log₂FC| > 0.25) because gene expression changes in peripheral blood samples are often attenuated compared to those in sorted cell populations or tissue biopsies, owing to signal dilution across heterogeneous circulating cell mixtures. This threshold is supported by methodological considerations from prior peripheral blood transcriptomic studies of asthma. Bjornsdottir et al. employed a similar cutoff (∼1.2-fold; |log₂FC| ≈ 0.26) in peripheral blood mononuclear cell (PBMC)-based asthma profiling, demonstrating that small but statistically significant changes in mixed cell populations can be biologically informative (20). In addition, Croteau-Chonka et al. showed that coordinated expression changes in whole blood, even when modest at the individual gene level, yield reproducible pathway-level signatures across 1,170 subjects (21). Persson et al. further demonstrated that cellular heterogeneity in pediatric peripheral blood leukocytes substantially influences gene-level expression estimates (22). Furthermore, biologically relevant genes such as BPGM and C-C motif chemokine ligand 2 (CCL2) would have been excluded at a more stringent threshold. To mitigate false-positive risk, we applied Benjamini-Hochberg correction for KEGG pathway enrichment and required independent concordance with metabolomic data through pathway intersection analysis. Nevertheless, the identified DEGs should be interpreted as candidate genes requiring independent validation.

### 2.4 Transcriptomic gene ontology (GO) and KEGG pathway enrichment analysis

GO and KEGG pathway enrichment analyses of the 15 differentially expressed genes were conducted using the clusterProfiler package in the R programming environment. Enrichment significance was calculated using the hypergeometric test with all annotated genes in the genome as the background set. Multiple testing correction was performed using the Benjamini-Hochberg method. Significantly enriched GO terms and KEGG pathways were identified using a significance threshold of adjusted *p* < 0.05.

### 2.5 Untargeted metabolomics analysis

Plasma samples from 15 normal-weight asthmatic children and 15 healthy control children at the First Affiliated Hospital of Xinjiang Medical University were collected for untargeted metabolite detection. The analysis was performed using a liquid chromatography-mass spectrometry (LC-MS) system. Samples were thawed at 4 °C and vortexed thoroughly. For each sample, 100 μL of plasma was mixed with 300 μL of methanol pre-chilled at −20 °C, vortexed, incubated at −20 °C for 1 h, and centrifuged at 2,300 × g, at 4 °C for 20 min. The supernatant was collected for LC-MS analysis. Throughout the analysis, samples were maintained at 4 °C in an autosampler. Samples were analyzed using a SHIMADZU Nexera X2 LC-30AD ultra-high performance liquid chromatography (UHPLC) system with an ACQUITY UPLC® HSS T3 column (2.1 × 100 mm, 1.8 µm, Waters, Milford, MA, USA). Chromatographic separation was performed under the following conditions: injection volume 16 μL; column temperature 40 °C; flow rate 0.3 mL/min; mobile phase A: 0.1% formic acid in water; mobile phase B: 0.1% formic acid in acetonitrile. Gradient elution program: 0.01–1 min, 2% B; 1–5 min, B increased linearly from 2% to 48%; 5–7 min, B increased linearly from 48% to 80%; 7–11 min, B increased linearly from 80% to 100%; 11–13 min, B maintained at 100%; 13–13.01 min, B decreased linearly from 100% to 2%; 13.01–15 min, B maintained at 2%.

Following UPLC separation, samples were analyzed using a Thermo Scientific Q Exactive Plus mass spectrometer. Each sample was analyzed in both positive (+) and negative (−) ion modes. The ionization was performed using electrospray ionization (ESI) with a Heated Electrospray Ionization (HESI) source. The ionization parameters were set as follows: spray voltage of 3.8 kV for positive ions and 3.2 kV for negative ions; capillary temperature of 320 °C; sheath gas flow rate of 30; auxiliary gas flow rate of 5; probe heater temperature of 350 °C; and S-Lens RF level of 50. Mass spectrometry acquisition parameters: acquisition time 15 min; precursor ion scan range 75–1,050 m/z; MS1 resolution 70,000 at m/z 200; automatic gain control (AGC) target 3 × 10^6^; maximum injection time (IT) 100 ms. MS2 spectra were acquired in data-dependent acquisition mode. After each full scan, MS2 spectra were triggered for the 10 most intense precursor ions with the following settings: MS2 resolution 17,500 at m/z 200; AGC target 1 × 10^5^; maximum IT 50 ms; activation type: higher-energy collisional dissociation (HCD); isolation window 2 m/z; normalized collision energy (stepped): 20, 30, and 40.

Raw data were processed for peak extraction and metabolite identification using MSDIAL software version 4.9. This process was based on accurate mass matching (mass deviation < 10 ppm) and MS/MS spectral matching (mass deviation < 0.01 Da), and involved searching public databases including HMDB, MassBank, GNPS, and the proprietary BaipuBio metabolite library (BP-DB). Furthermore, quality control (QC) samples were prepared by pooling equal volumes from all samples. System stability was evaluated through comparison of base peak chromatograms and principal component analysis (PCA). A total of 20,077 ion peaks were detected in both positive and negative ion modes. After removing ion peaks with >50% missing values within each group, data were normalized to the total peak area and preprocessed using unit variance (UV) scaling, followed by statistical analysis using the Python programming language. PCA was employed to assess overall sample separation trends and QC sample clustering. System stability was evaluated through seven-fold cross-validation. Orthogonal partial least squares discriminant analysis (OPLS-DA) was performed using R software, with R²Y > 0.5 and Q² > 0.5, indicating good explanatory and predictive capabilities of the model. Model quality was assessed using seven-fold cross-validation and tested for overfitting through 200 permutation tests. Differential metabolites were identified using the criteria of *p*-value < 0.05 and variable importance in projection (VIP) > 1. Hierarchical clustering analysis was performed on differential metabolites identified to visualize expression patterns. KEGG pathway enrichment analysis of differential metabolites was conducted using the clusterProfiler package in R, applying a significance threshold of *p* < 0.05. Statistical analyses were performed using Python version 3.9 (the scikit-learn package), with *p* < 0.05 considered statistically significant.

### 2.6 Multi-omics integration analysis

To identify core pathways co-regulated by transcriptomics and metabolomics, we performed an integrated analysis of KEGG enrichment results from both omics datasets. Significantly enriched KEGG pathways (*p* < 0.05) were extracted from transcriptomic and metabolomic analyses. These pathways were then matched using KEGG pathway IDs and intersected to identify shared pathways that were significantly enriched at both the transcriptional and metabolic levels. Differentially expressed genes and differentially abundant metabolites enriched in the intersected pathways were summarized, and the consistency of their up- or down-regulation patterns was analyzed.

### 2.7 Statistical analysis

All statistical analyses were performed using appropriate software packages. Clinical characteristics were compared using chi-square test for categorical variables and Mann–Whitney *U*-test for continuous variables (SPSS 29.0). Transcriptomic differential expression analysis used the limma package in R 4.5.2. Metabolomic data analysis employed Python 3.9 with scikit-learn package for PCA and OPLS-DA. Differential metabolites were identified using *p* < 0.05 and VIP > 1. KEGG pathway enrichment used clusterProfiler in R with *p* < 0.05 as significance threshold. Spearman rank correlation analysis was performed to evaluate the associations between the three key differential metabolites and clinical parameters [serum immunoglobulin E [IgE], eosinophil count, forced expiratory volume in 1 s percent predicted [FEV1%pred], and FEV_1_/forced vital capacity [FVC]] ratio in the combined cohort (*n* = 30), with results visualized as a correlation heatmap (23). Receiver operating characteristic (ROC) curves evaluated diagnostic performance, with area under the curve (AUC) > 0.7 indicating good performance and AUC > 0.8 indicating excellent performance. *p*-values < 0.05 were considered statistically significant for all analyses.

## 3 Results

### 3.1 Dataset sample characteristics

The GSE35571 dataset comprised 124 pediatric peripheral blood samples, including 60 asthmatic cases and 64 healthy controls. Subjects were aged 9–13 years and were from Detroit, Michigan, USA. After probe annotation and gene symbol mapping, 21,755 genes were retained for subsequent analysis (Table 1). Additionally, clinical data from our cohort included 15 normal-weight asthmatic children and 15 healthy control children, aged 6–14 years, with gender matching between groups.

**Table 1:** Differentially expressed genes between asthmatic and healthy children.

No.	Gene symbol	log₂FC	Average expression	*p*-value	Regulation
1	PRSS33	0.5319	6.65	0.0004	Up
2	OLIG2	0.2576	4.40	0.0013	Up
3	CEBPE	0.2627	6.21	0.0032	Up
4	CLC	0.4105	11.82	0.0037	Up
5	OLIG1	0.3404	8.44	0.0042	Up
6	PI3	0.5566	9.18	0.0068	Up
7	ADGRE1	0.2715	8.37	0.0071	Up
8	CCL2	−0.2886	3.14	0.0121	Down
9	RNASE3	0.3977	7.03	0.0139	Up
10	LOC102725068	0.4148	6.47	0.0169	Up
11	USP18	−0.2615	5.36	0.0287	Down
12	SLPI	0.3894	8.22	0.0342	Up
13	SCGB3A1	0.3039	5.73	0.0390	Up
14	BPGM	−0.2731	7.79	0.0422	Down
15	HEMGN	−0.2587	5.36	0.0496	Down

log₂FC > 0 indicates upregulation in asthmatic children; log₂FC < 0 indicates downregulation. FC, fold change.

### 3.2 Differential expression gene screening

**Figure 1:**
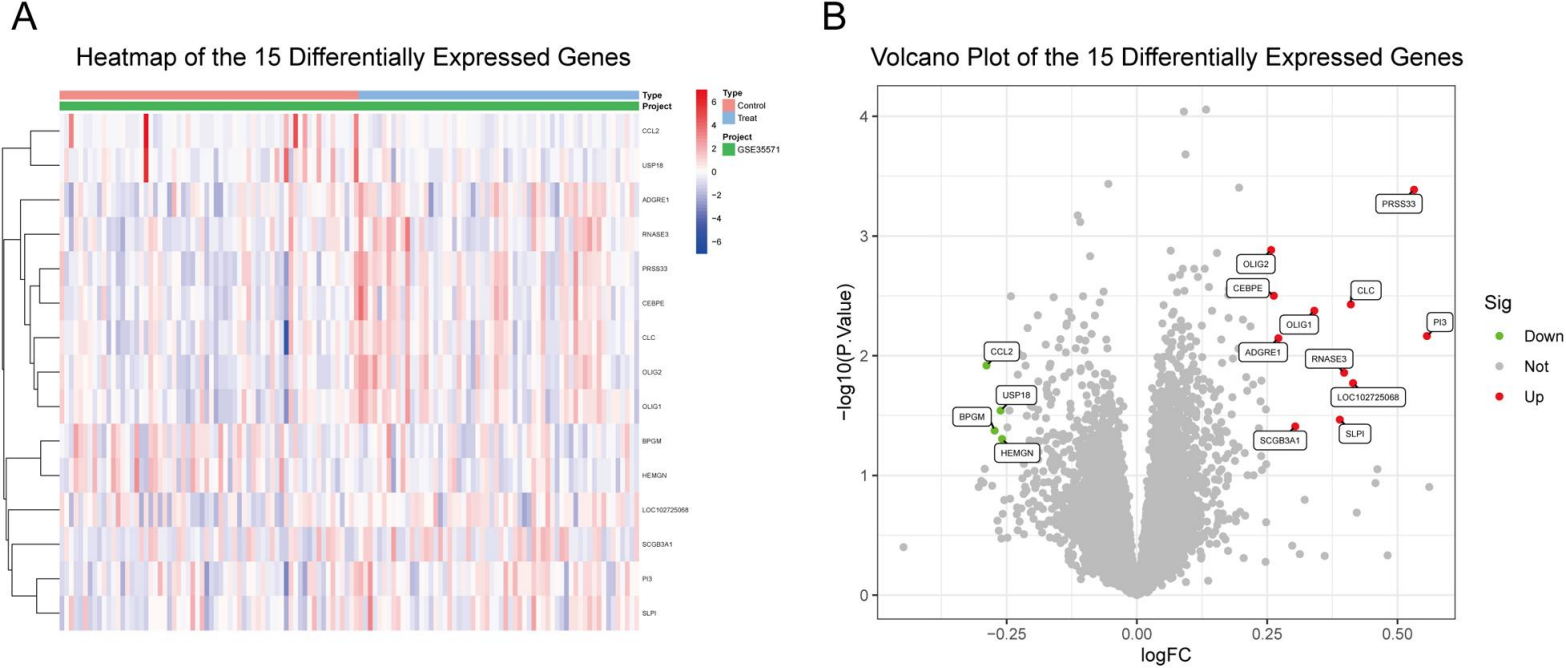
Differential gene expression analysis between asthmatic and healthy children. **(A)** Heatmap of 15 differentially expressed genes (DEGs). Red indicates high expression and blue indicates low expression. **(B)** Volcano plot showing the distribution of all genes. Red points indicate upregulated genes (*n* = 11), green points indicate downregulated genes (*n* = 4), and gray points indicate non-significant genes. DEGs were defined as *p* < 0.05 and |log₂FC| > 0.25.

During Differential expression analysis of the GSE35571 dataset (60 asthmatic patients vs. 64 healthy controls) was performed using the limma package in R software. Using criteria of *p* < 0.05 and | log_2_FC | > 0.25, we identified 15 differentially expressed genes, with detailed information presented in Table 1. Among these, 11 genes were upregulated in the asthma group, indicated by positive log_2_FC values, including CLC, RNASE3, CEBPE, PRSS33, PI3, SLPI, OLIG1, OLIG2, ADGRE1, SCGB3A1, and LOC102725068. Four genes were downregulated, indicated by negative log_2_FC values, including CCL2, USP18, BPGM, and HEMGN (Figures 1A,B). The upregulated genes were primarily involved in eosinophil activation (CLC, RNASE3, CEBPE), protease/antiprotease balance (PRSS33, PI3, SLPI), and immune cell markers (ADGRE1). The downregulated genes included the inflammatory chemokine CCL2 and metabolism-related genes (BPGM, HEMGN).

### 3.3 GO and KEGG pathway enrichment analysis of differential genes

**Figure 2:**
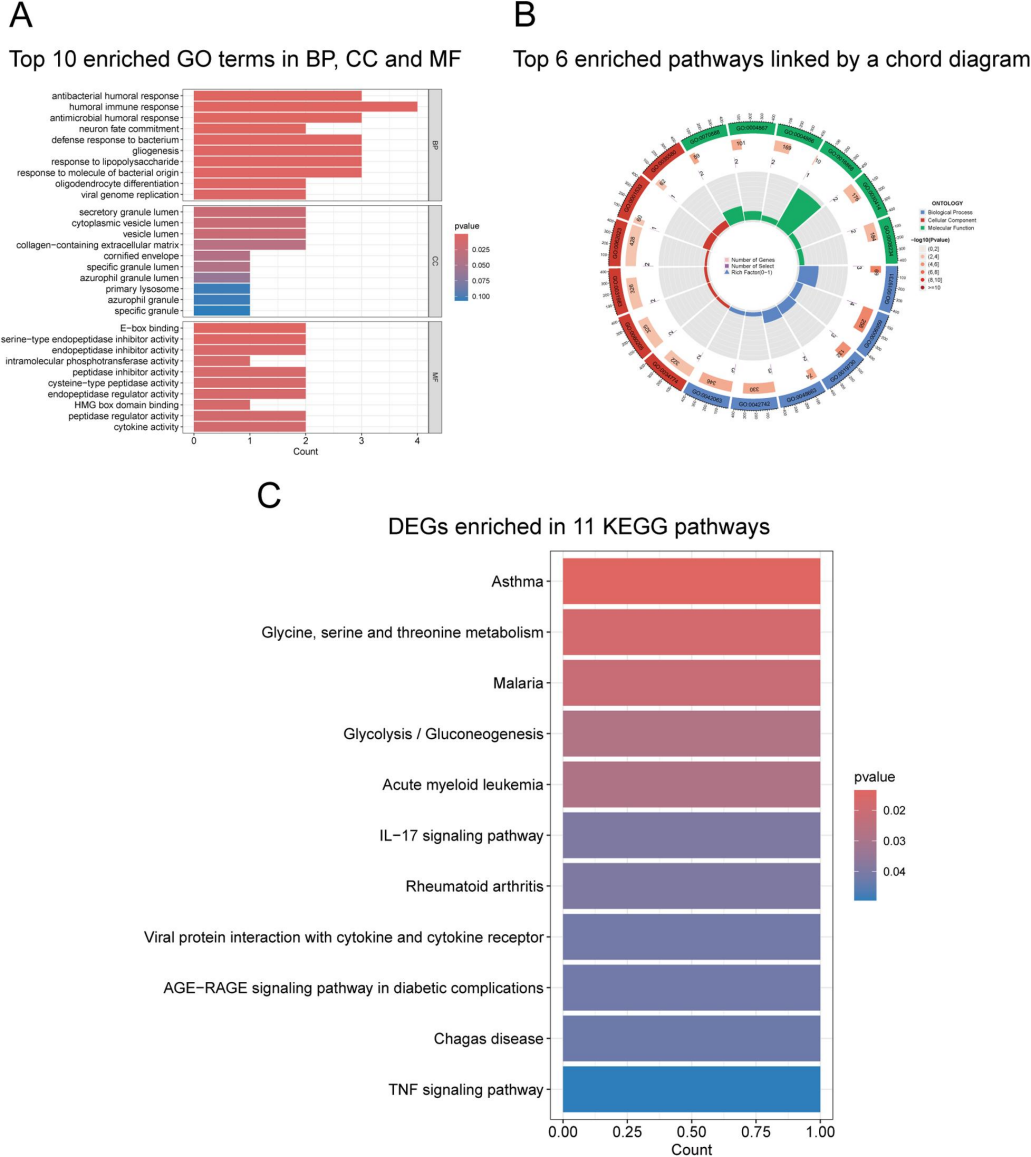
GO and KEGG pathway enrichment analysis of differentially expressed genes. **(A)** Bar plot showing the top 10 enriched GO terms in Biological Process (BP), Cellular Component (CC), and Molecular Function (MF) categories. The *x*-axis represents gene count and color indicates *P*-value. **(B)** Chord diagram illustrating the relationships between DEGs and the top 6 enriched GO terms per category. The outer arc represents GO terms (left) and genes (right), with connecting chords indicating gene-pathway associations. **(C)** Bar plot displaying 11 significantly enriched KEGG pathways (*p* < 0.05). Pathways are ranked by *p*-value in ascending order, with color gradient from red (lower *p*-value) to blue (higher *p*-value).

GO enrichment analysis of the 15 differentially expressed genes, using *p* < 0.05 as the screening criterion, identified 129 GO terms, including 106 biological processes (BP), 6 cellular components (CC), and 17 molecular functions (MF). Among these genes, twelve were successfully annotated; ADGRE1, HEMGN, and LOC102725068 were excluded due to lack of GO annotation. The top 10 GO terms with the smallest *p*-values in each category were visualized in a bar chart (Figure 2A). To clearly illustrate the correspondence between the differentially expressed genes and enriched GO terms while maintaining graphical readability, the top 6 GO terms from each category were selected and visualized using a chord diagram (Figure 2B). Regarding BP, the differentially expressed genes were primarily enriched in the humoral immune response (rich facto*r* = 0.016), antibacterial humoral response (rich facto*r* = 0.043), and gliogenesis. For CC, these genes were mainly localized to the secretory granule lumen and vesicle lumen. In terms of MF, they predominantly exhibited serine-type endopeptidase inhibitor activity and E-box binding activity. Detailed information on the 26 enriched GO terms is presented in Supplementary Table S1.

KEGG pathway enrichment analysis of the 15 DEGs revealed that 4 genes (RNASE3, CCL2, CEBPE, BPGM) were successfully annotated in the KEGG database. Using a significance threshold of *p* < 0.05 for pathway enrichment, 11 KEGG signaling pathways were identified (Figure 2C, Table 2). According to KEGG pathway classification, these enriched pathways were primarily grouped into four categories: (1) Human Diseases (*n* = 6), which included immune disease-related pathways such as Asthma (hsa05310) and Rheumatoid arthritis (hsa05323); infectious disease-related pathways such as Malaria (hsa05144) and Chagas disease (hsa05142); cancer-related pathways including Acute myeloid leukemia (hsa05221); and metabolic disease-related pathways such as the AGE-RAGE signaling pathway in diabetic complications (hsa04933). (2) Metabolism (*n* = 2), comprising Glycine, serine and threonine metabolism (hsa00260) in amino acid metabolism and Glycolysis/Gluconeogenesis (hsa00010) in carbohydrate metabolism. (3) Organismal Systems (*n* = 1), represented by the IL-17 signaling pathway (hsa04657). (4) Environmental Information Processing (*n* = 2), including Viral protein interaction with cytokine and cytokine receptor (hsa04061) and the TNF signaling pathway (hsa04668). The Asthma pathway (hsa05310) exhibited the highest statistical significance of enrichment (*p* = 0.0135). These results indicate that the enrichment of multiple immune and inflammation-related pathways supports the immuno-inflammatory nature of asthma.

**Table 2:** KEGG pathway enrichment analysis of differentially expressed genes.

KEGG ID	Pathway description	Count	*p*-value	Q-value
hsa05310	Asthma	1	0.0135	0.0497
hsa00260	Glycine, serine and threonine metabolism	1	0.0173	0.0497
hsa05144	Malaria	1	0.0210	0.0497
hsa00010	Glycolysis/Gluconeogenesis	1	0.0281	0.0497
hsa05221	Acute myeloid leukemia	1	0.0285	0.0497
hsa04657	IL-17 signaling pathway	1	0.0396	0.0497
hsa05323	Rheumatoid arthritis	1	0.0396	0.0497
hsa04061	Viral protein interaction with cytokine and cytokine receptor	1	0.0417	0.0497
hsa04933	AGE-RAGE signaling pathway in diabetic complications	1	0.0421	0.0497
hsa05142	Chagas disease	1	0.0429	0.0497
hsa04668	TNF signaling pathway	1	0.0495	0.0521

Count indicates the number of enriched genes in each pathway. Q-value represents the false discovery rate (FDR) adjusted *p*-value using the Benjamini-Hochberg method. Pathways with *p* < 0.05 were considered significantly enriched. KEGG, Kyoto Encyclopedia of Genes and Genomes.

### 3.4 Differential metabolite statistics and KEGG pathway enrichment analysis in metabolomics

**Figure 3:**
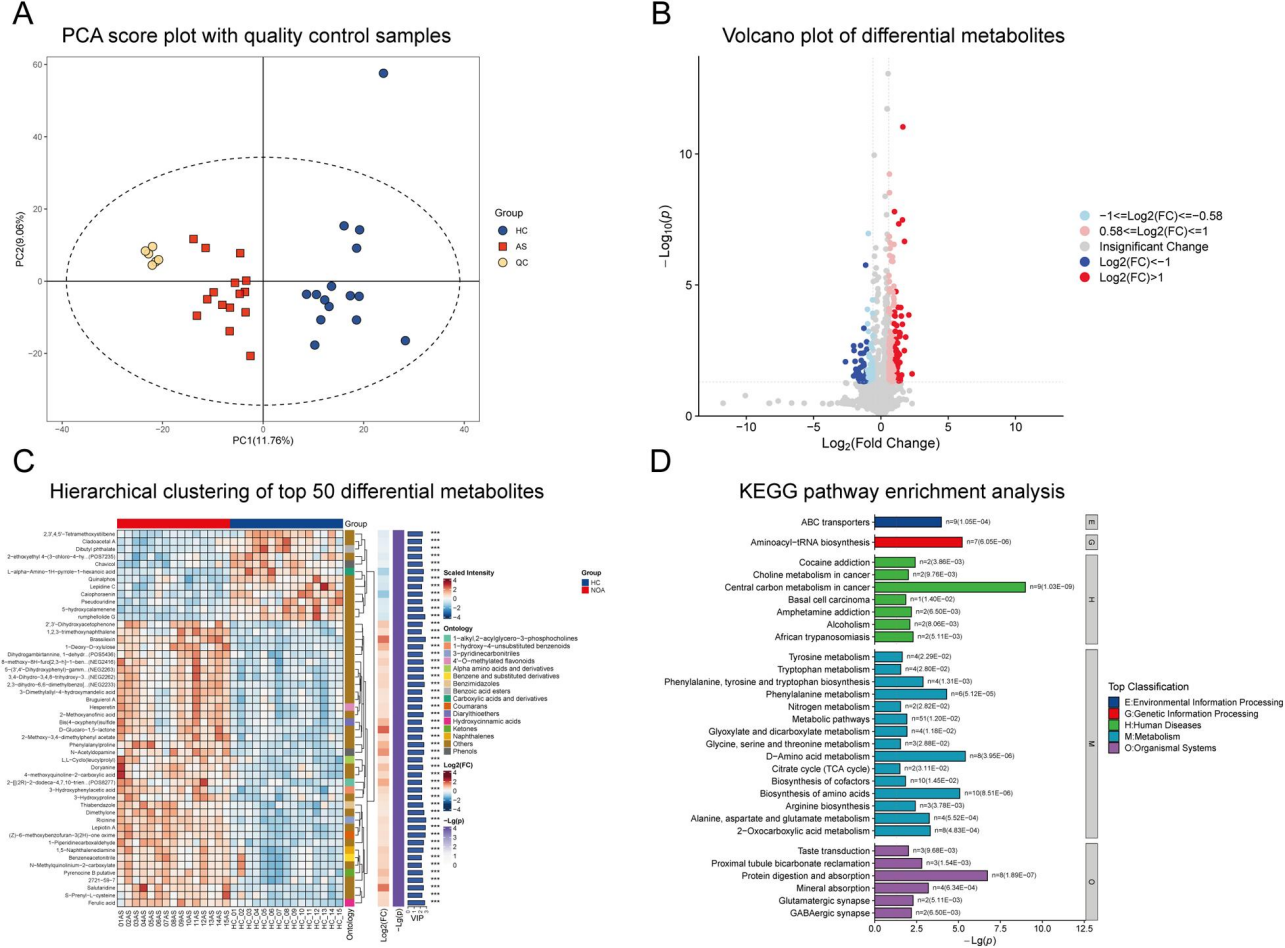
Untargeted metabolomics analysis of plasma samples from asthmatic and healthy children. **(A)** PCA score plot showing quality control (QC) samples clustered tightly, indicating reliable data quality. **(B)** Volcano plot displaying the distribution of differential metabolites. **(C)** Hierarchical clustering heatmap of top 50 differential metabolites. **(D)** KEGG pathway enrichment analysis of differential metabolites (Top 30).

The LC-MS platform detected a total of 1,994 metabolites (overall summary in Supplementary Supplementary Table S2). The PCA score plot showed that six QC samples clustered tightly, with center coordinates at PC1 = 5.9 and PC2 = −14.6, indicating good instrument stability and reliable data quality. Additionally, the asthma and control groups showed a clear separation along the PC2 axis (Figure 3A).

Using the criteria of *p* < 0.05 and VIP > 1, a total of 516 differential metabolites were identified, of which 373 (72.3%) were upregulated and 143 (27.7%) were downregulated. The volcano plot displayed the distribution of all metabolites. Significantly altered metabolites were color-coded according to fold change magnitude (Figure 3B). Hierarchical clustering analysis of the top 50 differential metabolites revealed distinctly different expression patterns between the two groups, with clear sample clustering separation. These metabolites belonged to 16 chemical classifications, including organic acids and derivatives, lipids and lipid-like molecules, and organoheterocyclic compounds (Figure 3C). KEGG pathway enrichment analysis identified 101 pathways. Among these, differential metabolites were significantly enriched in 34 pathways (*p* < 0.05). Detailed data are presented in Supplementary Supplementary Table S3, and the top 30 pathways are shown in Figure 3D. The pathways with the highest enrichment significance included Central Carbon Metabolism in Cancer (*n* = 9, *p* = 1.03 × 10^−9^), Protein Digestion and Absorption (*n* = 8, *p* = 1.89 × 10^−7^), D-Amino Acid Metabolism (*n* = 8, *P* = 3.95 × 10^−6^), Aminoacyl-tRNA Biosynthesis (*n* = 7, *p* = 6.05 × 10^−6^), and Biosynthesis of Amino Acids (*n* = 10, *p* = 8.51 × 10^−6^). Additionally, the Glycine, Serine, and Threonine Metabolism pathway (hsa00260) was also significantly enriched in the metabolomic analysis (*n* = 3, *p* = 0.0288).

### 3.5 Multi-Omics integration analysis identifies core intersection pathway

**Figure 4:**
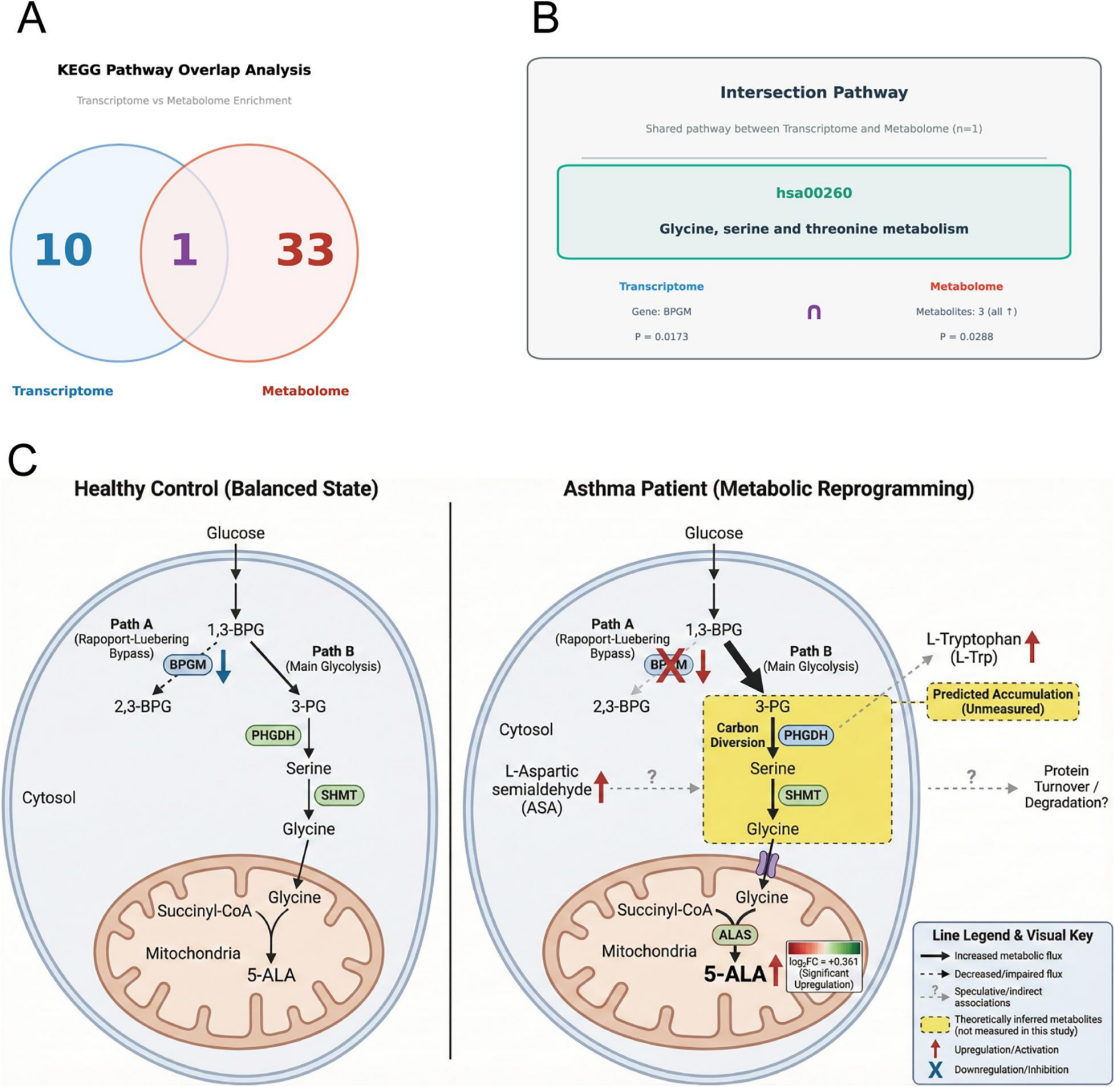
Multi-omics integration reveals BPGM-mediated metabolic reprogramming in childhood asthma **(A)** KEGG pathway enrichment overlap. Venn diagram showing one intersecting pathway (hsa00260: Glycine, serine and threonine metabolism) between transcriptomics and metabolomics analyses. **(B)** Intersection pathway details. The glycine, serine and threonine metabolism pathway was enriched in both omics datasets, with BPGM downregulation in transcriptomics and upregulation of three metabolites (5-ALA, ASA, and L-Trp) in metabolomics. **(C)** Putative metabolic mechanism. Left (Healthy controls): 1,3-BPG is metabolized through the Rapoport-Luebering shunt (BPGM-catalyzed, Path A) and main glycolytic pathway (Path B). 3-PG enters serine biosynthesis via PHGDH, followed by conversion to glycine (SHMT) and mitochondrial 5-ALA synthesis (ALAS). Right (Asthma patients): BPGM downregulation redirects carbon flux toward 3-PG accumulation (yellow highlight), potentially enhancing serine-glycine-5-ALA pathway activity. Serine and glycine levels were not measured; predicted changes (marked with “?”) are based on pathway inference. 5-ALA upregulation was confirmed by metabolomics. 1,3-BPG, 1,3-bisphosphoglycerate; 2,3-BPG, 2,3-bisphosphoglycerate; 3-PG, 3-phosphoglycerate; 5-ALA, 5-aminolevulinic acid; ALAS, 5-aminolevulinate synthase; ASA, L-aspartate semialdehyde; BPGM, bisphosphoglycerate mutase; KEGG, Kyoto Encyclopedia of Genes and Genomes; L-Trp, L-tryptophan; PHGDH, phosphoglycerate dehydrogenase; SHMT, serine hydroxymethyltransferase.

To comprehensively elucidate the molecular regulatory mechanisms of asthma, we integrated transcriptomic and metabolomic data for KEGG pathway intersection analysis. We identified the overlap between 11 significantly enriched KEGG pathways from transcriptomics and 34 significantly enriched pathways from metabolomics (all *p* < 0.05). Venn diagram analysis revealed one key shared pathway between both omics datasets (transcriptomics and metabolomics): hsa00260 (Glycine, serine, and threonine metabolism) (Figures 4A,B). Detailed intersection data are presented in Supplementary Supplementary Table S4.

This intersecting pathway was enriched with one differentially expressed gene, BPGM, in transcriptomics (*p* = 0.0173), and three differentially expressed metabolites in metabolomics: 5-aminolevulinic acid (5-ALA), L-aspartate semialdehyde (ASA), and L-tryptophan (L-Trp) (*p* = 0.0288). All three metabolites showed upregulation (Table 3).

**Table 3:** Intersection pathway between transcriptomics and metabolomics KEGG enrichment analysis.

Omics	Pathway ID	Pathway Name	Gene/Metabolite	*p*-value	Regulation
Transcriptomics	hsa00260	Glycine, serine and threonine metabolism	BPGM	0.0173	–
Metabolomics	hsa00260	Glycine, serine and threonine metabolism	5-Aminolevulinic acid	0.0288	↑
			L-Aspartate-semialdehyde		↑
			L-Tryptophan		↑

The intersection pathway hsa00260 (Glycine, serine and threonine metabolism) was identified by overlapping significantly enriched KEGG pathways (*p* < 0.05) from transcriptomics (11 pathways) and metabolomics (34 pathways). In transcriptomics, BPGM gene was enriched in this pathway. In metabolomics, three metabolites were enriched, all showing upregulation (↑) in the asthma group. BPGM, bisphosphoglycerate mutase.

### 3.6 Potential metabolic connection between BPGM downregulation and 5-ALA accumulation

Integration of transcriptomic and metabolomic data revealed a potential metabolic link between BPGM downregulation (log₂FC = −0.2731, *p* = 0.0173) and 5-ALA upregulation (log₂FC =  + 0.361, *p* < 0.05) in peripheral blood leukocytes (Supplementary Supplementary Table S5). BPGM, a key enzyme of the glycolytic Rapoport-Luebering shunt, catalyzes the conversion of 1,3-bisphosphoglycerate (1,3-BPG) to 2,3-bisphosphoglycerate (2,3-BPG). Its downregulation would theoretically redirect more 1,3-BPG into the main glycolytic pathway to generate 3-phosphoglycerate (3-PG), the direct precursor for serine biosynthesis. Based on established biochemical pathways, we hypothesize a potential metabolic cascade whereby 3-PG is oxidized by phosphoglycerate dehydrogenase (PHGDH) to initiate serine synthesis, serine is subsequently converted to glycine by serine hydroxymethyltransferase (SHMT), and glycine is then transported into the mitochondrial matrix where it condenses with succinyl-CoA to form 5-ALA via 5-aminolevulinate synthase (ALAS) (Figure 4C).

It should be noted that serine and glycine levels were not directly measured in this study; therefore, this proposed metabolic flux (3-PG → serine → glycine → 5-ALA) remains a hypothesis based on pathway analysis and existing literature, requiring validation through stable isotope tracing experiments. Additionally, the observed BPGM downregulation was detected in nucleated leukocytes rather than erythrocytes, suggesting that its function primarily involves cytosolic carbon flux redistribution rather than the erythrocyte-specific oxygen delivery regulation mediated by 2,3-BPG.

### 3.7 Correlation between key metabolites and clinical characteristics

**Figure 5:**
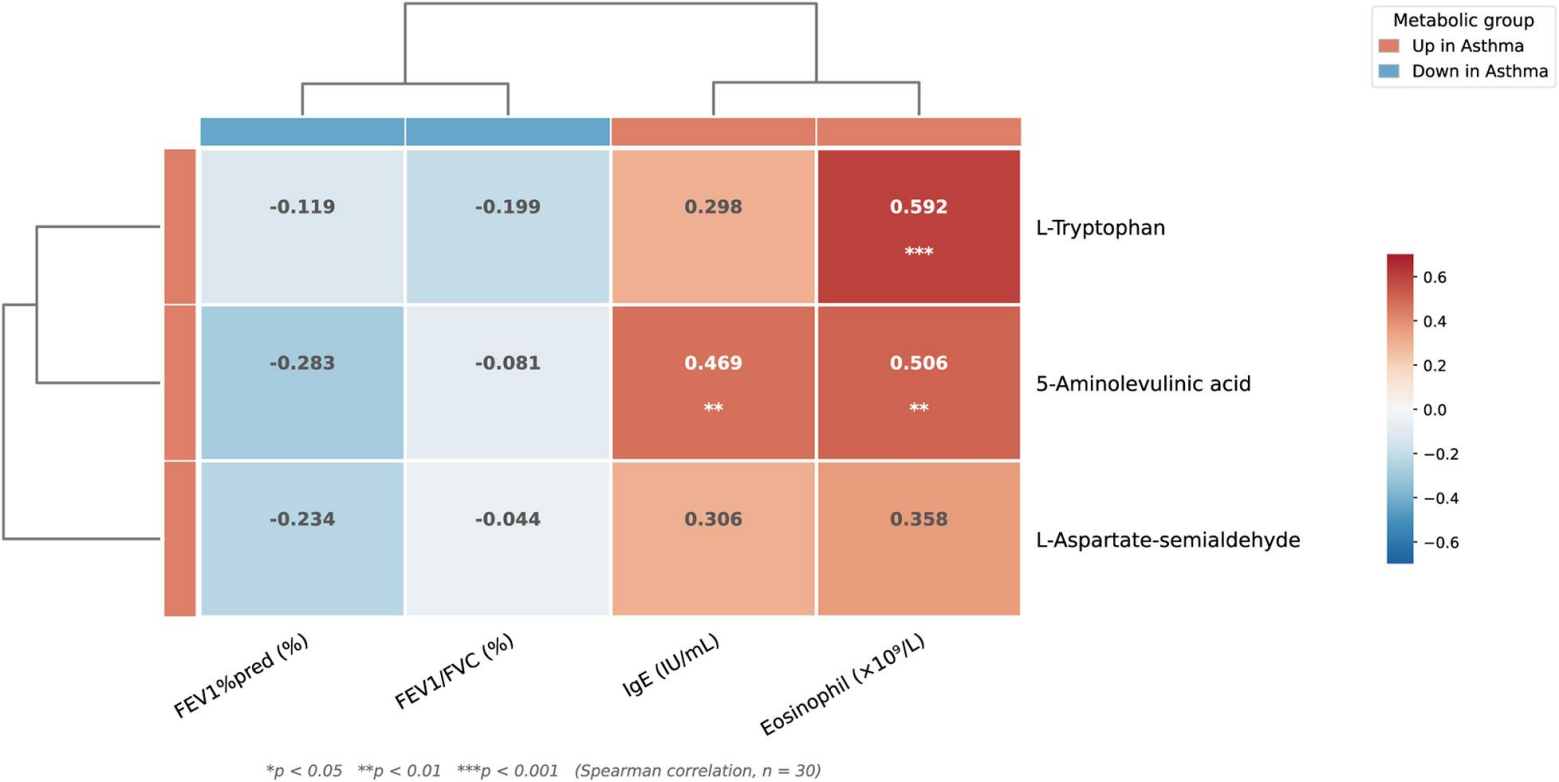
Correlation heatmap between key metabolites in the glycine-serine-threonine metabolism pathway and clinical characteristics. Spearman correlation coefficients between three key metabolites (L-Tryptophan, 5-Aminolevulinic acid, and L-Aspartate-semialdehyde) and clinical parameters (IgE, eosinophil count, FEV_1_%pred, and FEV_1_/FVC) in the combined cohort (*n* = 30). Hierarchical clustering dendrograms are shown on the left (metabolites) and top (clinical parameters). Color intensity within cells represents correlation strength; red indicates positive correlation and blue indicates negative correlation. Numerical values within cells represent Spearman r coefficients. The annotation color bars indicate the direction of change in asthma: red, upregulated in the asthma group; blue, downregulated in the asthma group. *p < 0.05, **p < 0.01, ***p < 0.001.

To further explore the association between metabolic phenotypes and clinical characteristics, Spearman correlation analysis was performed between the three key metabolites in the hsa00260 pathway and clinical parameters in the combined cohort (*n* = 30). The correlation heatmap revealed distinct patterns of association between metabolites and clinical indicators (Figure 5). Among the three metabolites, 5-aminolevulinic acid exhibited the most consistent correlations with inflammatory markers, showing significant positive correlations with both serum IgE levels (*r* = 0.469, *p* = 0.009) and peripheral blood eosinophil counts (*r* = 0.506, *p* = 0.004). L-Tryptophan demonstrated the strongest individual correlation, with a significant positive association with eosinophil counts (*r* = 0.592, *p* < 0.001), whereas its correlation with IgE levels did not reach statistical significance (*r* = 0.298, *p* = 0.110). L-Aspartate-semialdehyde showed a positive trend of correlation with eosinophil counts that approached but did not reach statistical significance (*r* = 0.358, *p* = 0.052). No significant correlations were observed between any of the three metabolites and pulmonary function parameters, including FEV1% predicted and FEV1/FVC ratio (all *p* > 0.05). Detailed correlation coefficients and *p*-values are presented in Supplementary Supplementary Table S6. These findings suggest that the identified metabolic alterations in the glycine-serine-threonine pathway are more closely associated with markers of allergic inflammation than with indices of airflow limitation.

### 3.8 Diagnostic value assessment of key metabolites in the intersection pathway

There were no significant differences between the two groups of children in terms of gender, age, weight, height, or BMI, which indicates good comparability of baseline characteristics. Serum IgE levels [ 206.51 (130.09, 357.65) vs. 29.54 (22.33, 53.11) IU/mL, *p* < 0.001] and peripheral blood eosinophil counts [0.46 (0.42, 0.58) vs. 0.23 (0.19, 0.26) × 10⁹/L, *p* < 0.001] were significantly higher in the asthma group compared to the control group. There were no significant differences in pulmonary function parameters between the groups: FEV_1_% predicted [96.40 (88.25, 108.55) vs. 105.20 (101.95, 108.50), *p* = 0.1013] and FEV_1_/FVC % [91.00 (80.95, 95.45) vs. 91.80 (90.90, 95.50), *p* = 0.2454]. Additionally, all three metabolite levels were significantly higher in the asthma group compared to the control group: L-Tryptophan [973.87 (716.66, 1,501.95) vs. 560.60 (454.72, 754.80), *p* = 0.0032], 5-Aminolevulinic acid [967.91 (917.22, 1,128.39) vs. 784.60 (672.90, 833.43), *p* < 0.001], and L-Aspartate-semialdehyde [1,313.21 (923.35, 1,573.39) vs. 856.63 (665.96, 1,013.98), *p* = 0.0032] (Table 4).

**Table 4:** Baseline characteristics comparison between two groups.

Variable	Asthma group (*n* = 15)	Control group(*n* = 15)	*p* value
Sex	7 (46.7%)/8 (53.3%)	7 (46.7%)/8 (53.3%)	1.0000
Age (years)	9.0 (8.0, 11.0)	9.0 (7.5, 12.0)	0.8836
Weight (kg)	31.0 (24.6, 36.4)	32.0 (26.0, 40.0)	0.8517
Height (m)	1.40 (1.31, 1.50)	1.42 (1.27, 1.55)	0.6178
BMI (kg/m²)	15.82 (14.56, 17.31)	16.02 (15.73, 16.60)	0.7400
IgE (IU/mL)	206.51 (130.09, 357.65)	29.54 (22.33, 53.11)	<0.001
Eosinophil count (×10⁹/L)	0.46 (0.42, 0.58)	0.23 (0.19, 0.26)	<0.001
FEV1%pred (%)	96.40 (88.25, 108.55)	105.20 (101.95, 108.50)	0.1013
FEV1/FVC (%)	91.00 (80.95, 95.45)	91.80 (90.90, 95.50)	0.2454
L-Tryptophan	973.87 (716.66, 1,501.95)	560.60 (454.72, 754.80)	0.0032
5-Aminolevulinic acid	967.91 (917.22, 1,128.39)	784.60 (672.90, 833.43)	<0.001
L-Aspartate-semialdehyde	1,313.21 (923.35, 1,573.39)	856.63 (665.96, 1,013.98)	0.0032

Categorical variables are presented as number (percentage) and compared using chi-square test; continuous variables are presented as median (interquartile range) and compared using Mann–Whitney *U*-test. All test statistics and *p* values are presented with four decimal places.

**Figure 6:**
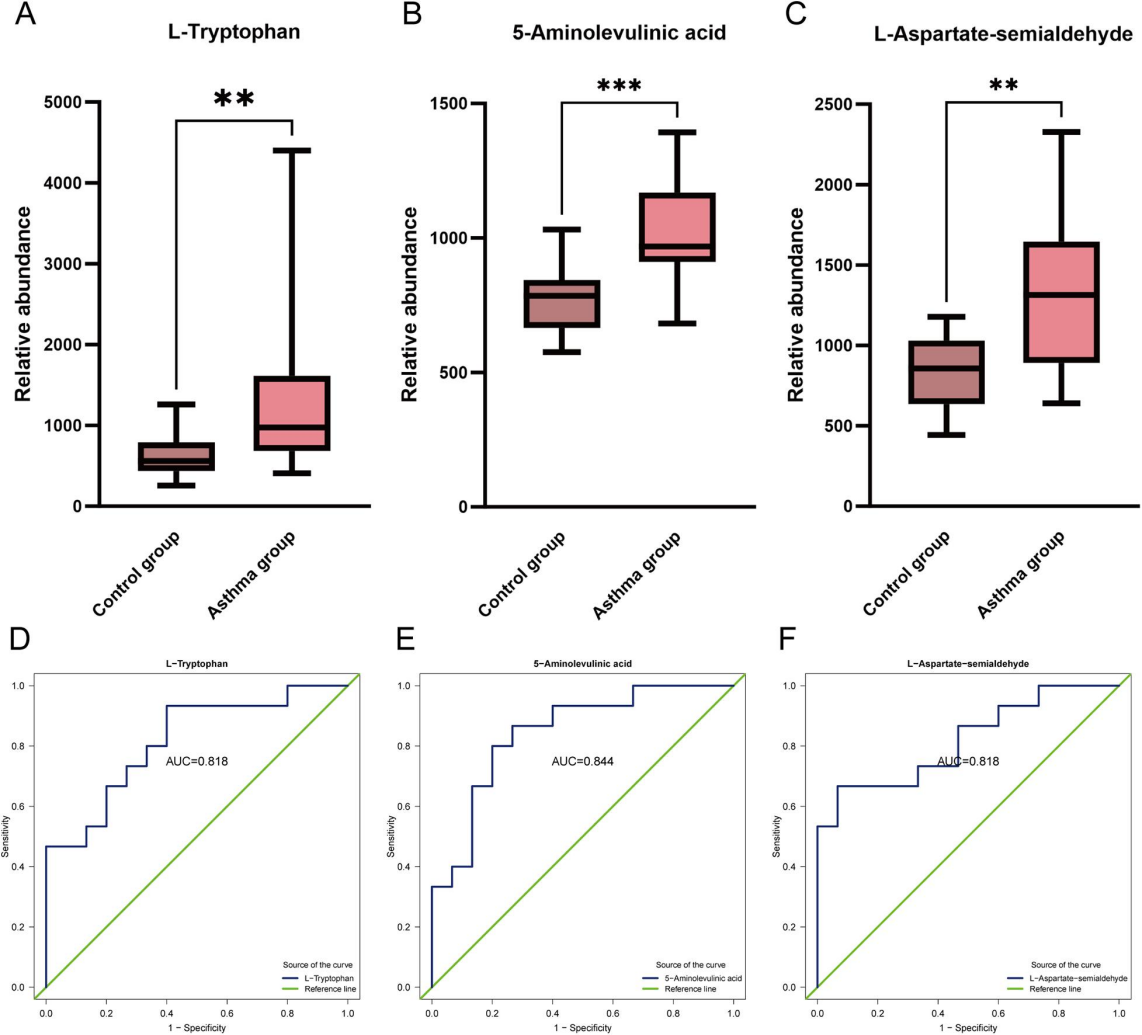
Differential expression and diagnostic performance of key metabolites in the glycine, serine and threonine metabolism pathway. **(A–C)** Box plots comparing relative abundance of L-Tryptophan, 5-Aminolevulinic acid, and L-Aspartate-semialdehyde between asthma and control groups. All metabolites were significantly elevated in asthma group (Mann–Whitney U test, **p < 0.01, ***p < 0.001). **(D–F)** ROC curves demonstrating diagnostic performance of the three metabolites. AUC values: L-Tryptophan = 0.818, 5-Aminolevulinic acid = 0.844, L-Aspartate-semialdehyde = 0.818. Green dashed line represents reference (AUC = 0.5). AUC, area under the curve; ROC, receiver operating characteristic.

ROC curve analysis demonstrated that the AUC values for the three metabolites were 0.818, 0.844, and 0.818, respectively, all exceeding 0.8, indicating good diagnostic accuracy (Figure 6). Among them, 5-Aminolevulinic acid exhibited the highest AUC (0.844), with both sensitivity and specificity of 80.0%, representing the most balanced diagnostic accuracy in terms of equal sensitivity and specificity.

## 4 Discussion

### 4.1 Multi-omics integration strategy and study design

The present study employed an integrated transcriptomics and metabolomics analysis strategy to explore the molecular characteristics of childhood asthma. This integrated analysis approach offers multiple advantages. First, the combined analysis of public transcriptomic data (GSE35571), with our in-house metabolomics cohort, constructed a putative molecular framework connecting gene expression patterns to metabolite alterations (24). Second, KEGG pathway intersection analysis significantly reduces false-positive risk. When the same pathway is independently identified by both transcriptomics and metabolomics, the combined false-positive probability is substantially lower than the threshold for single-omics studies (25). Third, untargeted metabolomics detected 1,994 metabolites with tight clustering of quality control samples, confirming data reliability (26). More importantly, this strategy not only validates known metabolic pathway abnormalities but also explores their upstream transcriptional regulatory mechanisms, which were not addressed in previous single-metabolomics studies (7).

Regarding the metabolomics cohort size (*n* = 15 per group), although this sample size may appear modest, it is consistent with established precedents in pediatric metabolomics research (27). Moreover, our metabolomics platform detected 1,994 metabolites with stringent quality control (tight QC clustering, PC1 = 5.9, PC2 = −14.6), thereby ensuring data reliability despite the sample size. The identification of 516 differential metabolites with clear group separation in PCA analysis further supports the robustness of our findings. Nevertheless, we acknowledge that larger validation cohorts are essential to confirm these exploratory findings and establish the clinical utility of the identified biomarkers, as discussed in the limitations section below. It should be noted that this cross-population integration design enables pathway-level discovery but cannot establish direct biological coupling between gene expression and metabolite changes within the same subjects. The observed pathway intersection on hsa00260 therefore represents indirect concordance — namely, the same pathway independently reaching statistical significance in two ethnically and geographically distinct populations — rather than direct evidence that BPGM expression regulates the identified metabolites within the same individuals. This approach is thus best suited for hypothesis generation, and the mechanistic interpretations proposed herein require validation through integrated multi-omics studies conducted within a single cohort.

### 4.2 BPGM downregulation and glycine-serine-threonine pathway dysregulation

This study identified BPGM downregulation in peripheral blood of children with asthma. BPGM encodes a key enzyme in the glycolytic Rapoport-Luebering shunt pathway, catalyzing the conversion of 1,3-BPG to 2,3-BPG (13). In erythrocytes, BPGM regulates hemoglobin oxygen affinity through modulation of 2,3-BPG concentration, and its deficiency can lead to tissue hypoxia and compensatory erythrocytosis (14). However, the association between BPGM and asthma has not been previously reported in the literature. Importantly, the transcriptomic data in this study were derived from peripheral blood samples, reflecting gene expression changes in nucleated leukocytes. While BPGM is predominantly expressed in erythrocytes, the gene is also expressed in leukocytes, where its function may differ from its classical erythrocyte role. BPGM downregulation may be associated with asthma pathophysiology through several possible mechanisms. First, decreased BPGM activity may reduce flux through the Rapoport-Luebering shunt, potentially redirecting glycolytic intermediates such as 3-PG toward alternative metabolic pathways (28). Second, 3-PG serves as the initial substrate for serine biosynthesis via PHGDH (17), which is subsequently converted to glycine by SHMT. In leukocytes with active mitochondria, glycine can condense with succinyl-CoA to form 5-ALA, the rate-limiting precursor of heme biosynthesis. This pathway could potentially explain the 5-ALA upregulation observed in our metabolomic data, which may reflect heme synthesis demand for proteins such as myeloperoxidase and cytochromes in inflammatory leukocytes. Third, BPGM downregulation may indicate broader metabolic alterations in inflammatory cells (5), suggesting that asthma involves both local airway inflammation and systemic metabolic changes. These findings provide insights into potential metabolic mechanisms in childhood asthma, though the proposed pathway requires validation through functional and mechanistic studies.

The significant enrichment of the glycine, serine, and threonine metabolism pathway (hsa00260) in this study is highly consistent with previous pediatric asthma metabolomics studies. Fitzpatrick et al. (11), and Cottrill et al. (12), successively confirmed the importance of this pathway in children with severe asthma and acute exacerbations, and the review by Park et al. (10) also summarized the trends in related metabolite changes. However, these studies primarily focused on metabolite description and lacked deeper molecular insights. Through multi-omics integration, the present study provides preliminary evidence suggesting a potential association between transcriptional changes and metabolic alterations in this pathway. While we observed concurrent upregulation of key metabolites and downregulation of the BPGM gene, the causal relationship between these changes remains to be established through functional studies.

Building on the mechanistic framework described above, BPGM downregulation may perturb the balance between glycolysis and serine/glycine metabolism through multiple mechanisms: First, reduced 2,3-BPG synthesis may alter glycolytic flux, affecting 3-PG availability for serine biosynthesis. Second, elevated serine-related metabolites (L-tryptophan, 5-aminolevulinic acid, L-aspartate semialdehyde) may represent compensatory responses to glycolytic stress. These findings suggest that BPGM may function as an upstream regulator in the glycolysis-amino acid metabolism axis, indicating a possible link between gene expression alterations and metabolic phenotype in asthma.

It should be emphasized that the proposed metabolic cascade (BPGM downregulation → 3-PG → serine → glycine → 5-ALA accumulation) is currently a theoretical hypothesis based on established biochemical pathways and our observational data. The levels of key intermediate metabolites, including serine and glycine, as well as the expression or activity of critical enzymes (PHGDH, SHMT, ALAS), were not directly measured in this study. Therefore, this proposed pathway should be considered as preliminary and hypothesis-generating, requiring rigorous experimental validation through stable isotope tracing, targeted metabolomics, and functional experiments such as BPGM knockdown/overexpression in leukocyte models before any mechanistic conclusions can be drawn.

A critical question is whether the enrichment of hsa00260 reflects asthma-specific metabolic dysregulation or merely a generalized inflammatory response. Several lines of evidence support the former interpretation. First, this pathway was the sole intersection between independently derived transcriptomic and metabolomic datasets, suggesting that its enrichment is not a nonspecific finding but rather a convergent signal identified through two orthogonal analytical approaches. Second, as discussed above, hsa00260 has been consistently implicated in pediatric asthma across multiple independent studies using different populations, sample types, and disease stages, which argues against a nonspecific inflammatory signature. Third, while other amino acid metabolism pathways were enriched in the metabolomic analysis alone—including aminoacyl-tRNA biosynthesis (*p* = 6.05 × 10⁻⁶), D-amino acid metabolism (*p* = 3.95 × 10⁻⁶), and biosynthesis of amino acids (*p* = 8.51 × 10⁻⁶)—none of these pathways reached significance in the transcriptomic enrichment analysis, and thus they did not appear in the cross-omics intersection. The near-significant enrichment of these pathways in metabolomics alone may reflect broader metabolic perturbation associated with the inflammatory state; however, their lack of transcriptomic support limits the evidence for their direct involvement in the gene-to-metabolite regulatory cascade explored in this study. We acknowledge that this does not exclude the possibility of broader metabolic reprogramming involving multiple pathways, and the specificity of hsa00260 to asthma vs. other inflammatory conditions remains to be determined through comparative studies.

### 4.3 Pathophysiological mechanisms in pediatric asthma

The metabolic alterations identified in this study may have particular significance in pediatric populations due to age-specific physiological characteristics. Children's rapidly developing immune systems and ongoing lung maturation (2) may render them particularly vulnerable to metabolic imbalances affecting inflammatory responses and oxidative stress pathways (11, 12). The dysregulation of glycine-serine-threonine metabolism during critical developmental windows could potentially influence the trajectory of asthma severity and treatment responsiveness. Furthermore, the observed BPGM downregulation may have implications for oxygen delivery efficiency during acute exacerbations when children's oxygen consumption is proportionally higher than adults. These pediatric-specific considerations underscore the importance of age-appropriate molecular research and the potential value of early metabolic intervention strategies. Differences between our study results and some international studies may reflect heterogeneity in population characteristics and disease phenotypes. Previous studies predominantly focused on children with severe asthma or acute exacerbations (11, 12). In contrast, the present study enrolled treatment-naive asthmatic children with normal weight—a population that better reflects the early metabolic characteristics of asthma onset rather than the metabolic alterations that occur following disease progression or therapeutic intervention. Additionally, our study population was from the Xinjiang region of China, while previous studies were primarily based on European and American populations; differences in genetic background and environmental exposures may influence metabolic phenotypes (9). For example, differential metabolites in this study were predominantly upregulated, whereas in Cottrill et al.'s study (12), upregulated and downregulated metabolites were relatively balanced. This difference may be related to disease severity, medication status, and ethnic differences. Despite these differences, the consistent enrichment of the hsa00260 pathway across different populations and disease stages reinforces its relevance to childhood asthma, as discussed above.

From a pathophysiological perspective, disruption of the glycine, serine, and threonine metabolism pathway (hsa00260) identified in this study may contribute to metabolic imbalance in asthma at multiple biological levels. One-carbon metabolism, a core component of this pathway, could potentially be affected. Serine provides one-carbon units that generate S-adenosylmethionine (SAM) through the folate cycle, serving as a universal methyl donor for epigenetic modifications (29). Therefore, altered serine metabolism may influence DNA methylation and histone modifications, potentially affecting transcriptional regulation of asthma-related genes. Second, this pathway is crucial for maintaining redox balance, as serine is an important precursor for synthesizing the major antioxidant glutathione. The metabolic products glycine and cysteine are essential for glutathione synthesis (30). Disruption of this pathway may compromise cellular antioxidant defense capacity in the context of oxidative stress associated with asthma. The upregulation of serine-related metabolites in this study may reflect the body's compensatory response to oxidative stress; however, under chronic asthmatic inflammatory conditions, such compensation is often insufficient, ultimately leading to airway epithelial damage and remodeling (11).

Furthermore, BPGM gene downregulation may exacerbate pathological processes by affecting erythrocyte function. 2,3-BPG, synthesized by BPGM catalysis, is a key molecule regulating hemoglobin oxygen affinity. Decreased levels enhance hemoglobin-oxygen binding, potentially limiting oxygen release to tissues (14). If such changes in oxygen-carrying function lead to tissue hypoxia, they may further activate hypoxia-inducible factor (HIF) signaling pathways, thereby amplifying inflammation and promoting airway remodeling (31). Additionally, the elevated levels of 5-aminolevulinic acid observed in this study, a precursor for heme synthesis (32), are also consistent with altered erythrocyte metabolic status. These metabolic alterations suggest that dysregulation in asthma may contribute to disease progression through systemic mechanisms such as interference with oxygenation and redox balance. Taken together, the above mechanisms are associated with the BPGM gene downregulation identified in this study; collectively, they suggest the potential existence of a systemic metabolic network disruption in asthma pathogenesis.

### 4.4 Clinical diagnostic biomarkers and advantages over conventional methods

The biomarkers identified in this study possess considerable clinical application potential. ROC curve analysis demonstrated that the AUC values for L-tryptophan, 5-aminolevulinic acid, and L-ASA were 0.818, 0.844, and 0.818, respectively; all met the criteria for good diagnostic performance. Among them, 5-aminolevulinic acid exhibited the best diagnostic performance. Moreover, these biomarkers may have potential for disease staging and phenotype specificities. The plasma metabolite biomarkers identified in this study offer several practical advantages over existing pediatric asthma diagnostic approaches. Spirometry, while essential for diagnosis and monitoring (19), requires patient cooperation and technical proficiency that may be challenging in children younger than 6 years. Fractional exhaled nitric oxide (FeNO) measurement, although non-invasive, demonstrates variable specificity and can be influenced by factors beyond eosinophilic inflammation (33). Bronchial challenge testing, considered the gold standard for assessing airway hyperresponsiveness, carries procedural risks and is not routinely performed in young children. In contrast, plasma biomarker measurement requires only a standard blood draw, is minimally invasive, and can be performed even in very young children or during acute illness. The excellent diagnostic performance observed for 5-aminolevulinic acid (AUC 0.844, 80% sensitivity/specificity) approaches clinically useful thresholds. Moreover, metabolite profiling provides mechanistic insights into disease pathophysiology that functional testing cannot reveal, potentially enabling phenotype-specific treatment selection. Future research should evaluate whether combining metabolite biomarkers with traditional diagnostic methods can improve early detection accuracy (34), particularly in preschool-aged children where diagnostic uncertainty is highest.

Correlation analysis between differential metabolites and clinical indicators provided additional context for interpreting the biological relevance of the identified metabolites. L-tryptophan showed the strongest individual correlation with eosinophil counts, suggesting a potential link between tryptophan metabolism and allergic inflammation. 5-Aminolevulinic acid demonstrated the most consistent pattern, with significant positive associations with both serum IgE and eosinophil counts. This finding may be biologically relevant given that eosinophil peroxidase is a heme-containing granule enzyme implicated in eosinophil-associated tissue injury (35). L-Aspartate-semialdehyde showed only a marginal trend, possibly reflecting its distal position in the metabolic network or limited statistical power. The absence of significant correlations with pulmonary function parameters may reflect the enrollment of treatment-naive children at initial diagnosis, in whom metabolic alterations may precede measurable airflow limitation. This analysis strategy is in line with recent metabolomic studies in allergic airway inflammation that correlated differential metabolites with clinical and immune indicators to enhance biological interpretation (23). However, these correlations should be interpreted cautiously given the modest sample size, and larger studies are needed to confirm these associations.

### 4.5 Implications for precision medicine and phenotype-based treatment

Beyond their diagnostic utility, these metabolites provide insights into distinct metabolic subtypes of childhood asthma. L-aspartate-semialdehyde, as a key intermediate in amino acid metabolism, directly participates in the lysine biosynthesis pathway (hsa00260) and may be more suitable than other metabolites as a specific marker for metabolic abnormality-associated asthma subtypes (36). 5-aminolevulinic acid is involved in heme synthesis and BPGM function; its elevated levels may reflect erythrocyte metabolic stress, indicating the presence of systemic metabolic imbalance (32). From a precision medicine perspective, combining BPGM gene expression levels with these biomarkers may potentially help explore asthma subtypes in future studies. This approach may provide a preliminary framework for future investigation of individualized treatment strategies, pending validation (37). Children with low BPGM expression combined with metabolite abnormalities may represent an asthma subtype with distinct metabolic features, a hypothesis supported by observed metabolic profiles and providing a theoretical basis for future exploration of individualized treatment based on metabolic phenotypes. Looking forward, should these findings be validated in larger independent cohorts, integrating BPGM expression profiling with metabolite panel testing could potentially contribute to precision medicine approaches in pediatric asthma management. However, this prospect remains highly preliminary given the exploratory nature of the current study.

### 4.6 Limitations

However, this study has several limitations. First, the metabolomics sample size is modest (*n* = 15 per group), requiring validation in larger cohorts despite stringent quality control. Additionally, the |log₂FC| threshold of 0.25 used for DEG screening is lower than conventional thresholds typically applied to tissue-based transcriptomic studies. Although this threshold is supported by precedent in peripheral blood studies, where expression differences are typically attenuated by cellular heterogeneity and small but statistically significant changes may still be biologically informative (20), it may increase the false-positive rate. The biological significance of the identified DEGs, particularly BPGM, therefore requires confirmation through independent cohort validation and functional assays. Second, and most critically, the cross-population integration design constitutes the principal methodological limitation of this study. The transcriptomic data were derived from a U.S. cohort (Detroit, Michigan), while the metabolomic data were obtained from a Chinese cohort (Xinjiang, China). This design precludes direct gene–metabolite correlation analysis at the individual level. The two cohorts may differ substantially in genetic background, dietary patterns, environmental exposures, and gut microbiome composition, all of which can independently influence metabolic profiles. Population-specific genetic variants may vary markedly in allele frequency across ethnic groups and contribute to differences in metabolic traits, as exemplified by the CREBRF variant identified in Samoan populations, which is rare in other populations and has a substantial impact on energy metabolism (38). For example, traffic-related air pollution has been shown to elicit measurable perturbations in plasma metabolomic signatures through oxidative stress and inflammatory pathways (39), and such environmental exposures differ markedly between Detroit and Xinjiang. Additionally, gut microbiota composition varies significantly across ethnic groups even within the same geographic setting and is only partly explained by lifestyle or dietary factors (40), suggesting that population-specific microbial communities may independently contribute to metabolomic differences. Although pathway-level intersection analysis may reduce population-specific confounding by focusing on conserved biological processes, it cannot exclude the possibility that similar pathway enrichment arises from distinct underlying mechanisms in the two cohorts. Furthermore, the absence of matched transcriptomic and metabolomic data from the same individuals means that the proposed BPGM-mediated metabolic cascade remains inferential and not supported by within-subject validation. Future studies should prioritize the collection of paired transcriptomic and metabolomic data from the same individuals, ideally within multi-ethnic cohorts, to enable direct gene–metabolite correlation analyses and to validate the cross-population findings reported here. Third, the cross-sectional design cannot establish temporal relationships or assess predictive value for disease progression. Fourth, findings may not generalize to obesity-associated asthma or other phenotypes. Fifth, the mechanistic interpretation linking BPGM downregulation to downstream metabolite alterations remains largely theoretical. The proposed metabolic cascade was inferred from known biochemical pathways and pathway enrichment analysis, but key intermediate metabolites and the expression levels of critical enzymes were not directly measured. Without stable isotope tracing or targeted enzyme activity assays, the actual direction and magnitude of metabolic flux through this pathway cannot be confirmed. Sixth, the clinical translation of the identified biomarkers faces several constraints. The diagnostic performance was evaluated in a small, single-center cohort without external validation. The metabolites identified are not specific to asthma and may be elevated in other inflammatory or metabolic conditions. Furthermore, untargeted metabolomics provides semi-quantitative data; absolute quantification through targeted assays and the establishment of clinically applicable cutoff values are prerequisites for any diagnostic application. Cost-effectiveness analyses and integration with existing clinical workflows remain to be investigated before these biomarkers can be considered for routine clinical use.

## 5 Conclusions

This multi-omics integration study identified BPGM downregulation in peripheral blood leukocytes of children with asthma and revealed concurrent dysregulation of glycine-serine-threonine metabolism through convergent transcriptomic and metabolomic analysis. Integration of gene expression, pathway enrichment, and metabolite profiling suggests a putative metabolic pathway linking BPGM downregulation to altered metabolite profiles, indicating potential systemic metabolic alterations that may extend beyond local airway inflammation. Three metabolites showed promising diagnostic potential, particularly 5-ALA with optimal accuracy. These preliminary findings provide initial clues regarding potential metabolic-transcriptional associations in childhood asthma. However, the proposed mechanistic links are based on pathway-level inference and indirect evidence; therefore, these results should be regarded as hypothesis-generating rather than confirmatory. Future studies employing stable isotope tracing, targeted enzyme activity assays, and paired multi-omics analysis within the same cohort are essential to validate these observations and assess their clinical relevance.

## Data Availability

The transcriptomic data analyzed in this study are publicly available in the NCBI Gene Expression Omnibus (GEO) database under accession number GSE35571 (https://www.ncbi.nlm.nih.gov/geo/query/acc.cgi?acc=GSE35571). The metabolomics data generated in this study have been deposited in the Figshare repository, accessible via DOI: 10.6084/m9.figshare.32096113 (https://doi.org/10.6084/m9.figshare.32096113).
