# Supplementary material for: Multi-omics integration reveals BPGM downregulation and potential plasma metabolite biomarkers for childhood asthma
Source: Front Pediatr. 2026 May 7;14:1794811. doi: 10.3389/fped.2026.1794811 (PMC13189880; doi:10.3389/fped.2026.1794811)
Supplement: Supplementary file 1 [file Table1.docx]

Supplementary Material

## Supplementary Tables

**Supplementary Table S1. Gene Ontology enrichment analysis of differentially expressed genes**

| **GO ID** | **Description** | **Count** | **Rich Factor** | **Fold Enrichment** | **zScore** | ***p*-value** | ***p*.adjust** | **Genes** |
| --- | --- | --- | --- | --- | --- | --- | --- | --- |
| ***Biological Process (BP)*** | | | | | | | | |
| 0019731 | Antibacterial humoral response | 3 | 0.0435 | 58.6600 | 13.0700 | 0.0000 | 0.0052 | PI3/RNASE3/SLPI |
| 0006959 | Humoral immune response | 4 | 0.0155 | 20.9200 | 8.7700 | 0.0000 | 0.0052 | PI3/CCL2/RNASE3/SLPI |
| 0019730 | Antimicrobial humoral response | 3 | 0.0227 | 30.6600 | 9.3100 | 0.0001 | 0.0130 | PI3/RNASE3/SLPI |
| 0048663 | Neuron fate commitment | 2 | 0.0270 | 36.4600 | 8.3200 | 0.0013 | 0.0943 | OLIG2/OLIG1 |
| 0042742 | Defense response to bacterium | 3 | 0.0091 | 12.2600 | 5.6200 | 0.0017 | 0.0943 | PI3/RNASE3/SLPI |
| 0042063 | Gliogenesis | 3 | 0.0087 | 11.7000 | 5.4700 | 0.0019 | 0.0943 | OLIG2/OLIG1/CCL2 |
| 0032496 | Response to lipopolysaccharide | 3 | 0.0086 | 11.6300 | 5.4500 | 0.0019 | 0.0943 | CEBPE/CCL2/SLPI |
| 0002237 | Response to molecule of bacterial origin | 3 | 0.0081 | 10.9700 | 5.2700 | 0.0023 | 0.0975 | CEBPE/CCL2/SLPI |
| 0048709 | Oligodendrocyte differentiation | 2 | 0.0189 | 25.4600 | 6.8800 | 0.0027 | 0.1026 | OLIG2/OLIG1 |
| 0019079 | Viral genome replication | 2 | 0.0155 | 20.9200 | 6.1800 | 0.0040 | 0.1311 | CCL2/SLPI |
| ***Cellular Component (CC)*** | | | | | | | | |
| 0034774 | Secretory granule lumen | 2 | 0.0062 | 8.8300 | 3.7600 | 0.0209 | 0.0927 | RNASE3/SLPI |
| 0060205 | Cytoplasmic vesicle lumen | 2 | 0.0062 | 8.7400 | 3.7400 | 0.0213 | 0.0927 | RNASE3/SLPI |
| 0031983 | Vesicle lumen | 2 | 0.0061 | 8.7200 | 3.7300 | 0.0214 | 0.0927 | RNASE3/SLPI |
| 0062023 | Collagen-containing extracellular matrix | 2 | 0.0047 | 6.6400 | 3.1300 | 0.0354 | 0.0927 | CLC/SLPI |
| 0001533 | Cornified envelope | 1 | 0.0167 | 23.6800 | 4.6700 | 0.0414 | 0.0927 | PI3 |
| 0035580 | Specific granule lumen | 1 | 0.0161 | 22.9200 | 4.5900 | 0.0428 | 0.0927 | SLPI |
| ***Molecular Function (MF)*** | | | | | | | | |
| 0070888 | E-box binding | 2 | 0.0339 | 44.8500 | 9.2800 | 0.0009 | 0.0310 | OLIG2/OLIG1 |
| 0004867 | Serine-type endopeptidase inhibitor activity | 2 | 0.0198 | 26.2000 | 6.9800 | 0.0026 | 0.0422 | PI3/SLPI |
| 0004866 | Endopeptidase inhibitor activity | 2 | 0.0118 | 15.6600 | 5.2600 | 0.0070 | 0.0422 | PI3/SLPI |
| 0016868 | Intramolecular phosphotransferase activity | 1 | 0.1000 | 132.3000 | 11.4200 | 0.0075 | 0.0422 | BPGM |
| 0030414 | Peptidase inhibitor activity | 2 | 0.0114 | 15.0300 | 5.1500 | 0.0076 | 0.0422 | PI3/SLPI |
| 0008234 | Cysteine-type peptidase activity | 2 | 0.0109 | 14.3800 | 5.0200 | 0.0083 | 0.0422 | CLC/USP18 |
| 0061135 | Endopeptidase regulator activity | 2 | 0.0108 | 14.2300 | 4.9900 | 0.0084 | 0.0422 | PI3/SLPI |
| 0071837 | HMG box domain binding | 1 | 0.0769 | 101.7700 | 10.0000 | 0.0098 | 0.0428 | OLIG2 |
| 0061134 | Peptidase regulator activity | 2 | 0.0086 | 11.4100 | 4.3900 | 0.0129 | 0.0473 | PI3/SLPI |
| 0005125 | Cytokine activity | 2 | 0.0084 | 11.1200 | 4.3200 | 0.0135 | 0.0473 | CCL2/SCGB3A1 |

Note: Fold Enrichment indicates the enrichment magnitude. P.adjust represents the Benjamini-Hochberg adjusted *p*-value. Top 10 terms for BP and MF, and all 6 significant CC terms (*p* < 0.05) are shown. GO, Gene Ontology; BP, biological process; CC, cellular component; MF, molecular function. Rich Factor = number of enriched genes / number of background genes in the pathway.

**Supplementary Table S2. Summary of metabolomics analysis**

| **Item** | **Value** | **Description** |
| --- | --- | --- |
| Detection platform | LC-MS | Untargeted metabolomics |
| Total samples | 30 | Asthma (n = 15) + Control (n = 15) |
| Total detected metabolites | 1,994 | Positive and negative ion modes |
| Differential metabolites (P < 0.05, VIP > 1) | 516 | Dual screening by P-value and VIP |
| KEGG pathways mapped | 101 | Pathways annotated by detected metabolites |
| Upregulated metabolites | 373 (72.3%) | Higher in asthma group |
| Downregulated metabolites | 143 (27.7%) | Lower in asthma group |
| Log₂FC range | -10 to 10 | Fold change magnitude |
| VIP range | 1 to 2 | Variable Importance in Projection |
| Major chemical classes |  |  |
| Lipids and lipid-like molecules | 22.7% | Primary class |
| Organoheterocyclic compounds | 15.7% |  |
| Organic acids and derivatives | 15.1% |  |
| Enriched KEGG pathways (P < 0.05) | 34 | Significantly enriched pathways |

Note: LC-MS, liquid chromatography-mass spectrometry; VIP, Variable Importance in Projection; FC, fold change; KEGG, Kyoto Encyclopedia of Genes and Genomes. Differential metabolites were screened using the criteria of *p* < 0.05 and VIP > 1. Data were obtained from untargeted metabolomics analysis of plasma samples from 30 subjects (15 normal-weight asthmatic children and 15 healthy controls).

**Supplementary Table S3. KEGG pathway enrichment analysis of differential metabolites**

| **ID** | **Description** | **Metabo Ratio** | **Rich Factor** | **Fold Enrichment** | ***p*-value** | ***p*.adjust** | **Count** | **Class** | **Up** | **Down** |
| --- | --- | --- | --- | --- | --- | --- | --- | --- | --- | --- |
| hsa05230 | Central carbon metabolism in cancer | 9/62 | 0.2432 | 17.3919 | 1.03×10⁻⁹ | 1.04×10⁻⁷ | 9 | H | 7 | 2 |
| hsa04974 | Protein digestion and absorption | 8/62 | 0.1702 | 12.1702 | 1.89×10⁻⁷ | 9.54×10⁻⁶ | 8 | O | 6 | 2 |
| hsa00470 | D-Amino acid metabolism | 8/62 | 0.1159 | 8.2899 | 3.95×10⁻⁶ | 0.0001 | 8 | M | 6 | 2 |
| hsa00970 | Aminoacyl-tRNA biosynthesis | 7/62 | 0.1346 | 9.6250 | 6.05×10⁻⁶ | 0.0002 | 7 | G | 6 | 1 |
| hsa01230 | Biosynthesis of amino acids | 10/62 | 0.0781 | 5.5859 | 8.51×10⁻⁶ | 0.0002 | 10 | M | 8 | 2 |
| hsa00360 | Phenylalanine metabolism | 6/62 | 0.1224 | 8.7551 | 5.12×10⁻⁵ | 0.0009 | 6 | M | 4 | 2 |
| hsa02010 | ABC transporters | 9/62 | 0.0652 | 4.6630 | 0.0001 | 0.0015 | 9 | E | 7 | 2 |
| hsa01210 | 2-Oxocarboxylic acid metabolism | 8/62 | 0.0597 | 4.2687 | 0.0005 | 0.0061 | 8 | M | 7 | 1 |
| hsa00250 | Alanine, aspartate and glutamate metabolism | 4/62 | 0.1429 | 10.2143 | 0.0006 | 0.0062 | 4 | M | 4 | 0 |
| hsa04978 | Mineral absorption | 4/62 | 0.1379 | 9.8621 | 0.0006 | 0.0064 | 4 | O | 3 | 1 |
| hsa00400 | Phenylalanine, tyrosine and tryptophan biosynthesis | 4/62 | 0.1143 | 8.1714 | 0.0013 | 0.0121 | 4 | M | 4 | 0 |
| hsa04964 | Proximal tubule bicarbonate reclamation | 3/62 | 0.1765 | 12.6176 | 0.0015 | 0.0130 | 3 | O | 2 | 1 |
| hsa00220 | Arginine biosynthesis | 3/62 | 0.1304 | 9.3261 | 0.0038 | 0.0279 | 3 | M | 2 | 1 |
| hsa05030 | Cocaine addiction | 2/62 | 0.2857 | 20.4286 | 0.0039 | 0.0279 | 2 | H | 2 | 0 |
| hsa04724 | Glutamatergic synapse | 2/62 | 0.2500 | 17.8750 | 0.0051 | 0.0322 | 2 | O | 2 | 0 |
| hsa05143 | African trypanosomiasis | 2/62 | 0.2500 | 17.8750 | 0.0051 | 0.0322 | 2 | H | 2 | 0 |
| hsa04727 | GABAergic synapse | 2/62 | 0.2222 | 15.8889 | 0.0065 | 0.0365 | 2 | O | 2 | 0 |
| hsa05031 | Amphetamine addiction | 2/62 | 0.2222 | 15.8889 | 0.0065 | 0.0365 | 2 | H | 2 | 0 |
| hsa05034 | Alcoholism | 2/62 | 0.2000 | 14.3000 | 0.0081 | 0.0428 | 2 | H | 2 | 0 |
| hsa04742 | Taste transduction | 3/62 | 0.0938 | 6.7031 | 0.0097 | 0.0469 | 3 | O | 2 | 1 |
| hsa05231 | Choline metabolism in cancer | 2/62 | 0.1818 | 13.0000 | 0.0098 | 0.0469 | 2 | H | 2 | 0 |
| hsa00630 | Glyoxylate and dicarboxylate metabolism | 4/62 | 0.0625 | 4.4688 | 0.0118 | 0.0528 | 4 | M | 3 | 1 |
| hsa01100 | Metabolic pathways | 51/62 | 0.0167 | 1.1964 | 0.0120 | 0.0528 | 51 | M | 38 | 13 |
| hsa05217 | Basal cell carcinoma | 1/62 | 1.0000 | 71.5000 | 0.0140 | 0.0585 | 1 | H | 1 | 0 |
| hsa01240 | Biosynthesis of cofactors | 10/62 | 0.0305 | 2.1799 | 0.0145 | 0.0585 | 10 | M | 8 | 2 |
| hsa00350 | Tyrosine metabolism | 4/62 | 0.0513 | 3.6667 | 0.0229 | 0.0890 | 4 | M | 3 | 1 |
| hsa00380 | Tryptophan metabolism | 4/62 | 0.0482 | 3.4458 | 0.0280 | 0.1000 | 4 | M | 3 | 1 |
| hsa00910 | Nitrogen metabolism | 2/62 | 0.1053 | 7.5263 | 0.0282 | 0.1000 | 2 | M | 2 | 0 |
| hsa00260 | Glycine, serine and threonine metabolism | 3/62 | 0.0625 | 4.4688 | 0.0288 | 0.1000 | 3 | M | 3 | 0 |
| hsa00020 | Citrate cycle (TCA cycle) | 2/62 | 0.1000 | 7.1500 | 0.0311 | 0.1050 | 2 | M | 1 | 1 |
| hsa00232 | Caffeine metabolism | 2/62 | 0.0909 | 6.5000 | 0.0372 | 0.1210 | 2 | M | 1 | 1 |
| hsa05211 | Renal cell carcinoma | 1/62 | 0.3333 | 23.8333 | 0.0414 | 0.1310 | 1 | H | 0 | 1 |
| hsa04976 | Bile secretion | 4/62 | 0.0412 | 2.9485 | 0.0458 | 0.1390 | 4 | O | 3 | 1 |
| hsa01232 | Nucleotide metabolism | 3/62 | 0.0517 | 3.6983 | 0.0467 | 0.1390 | 3 | M | 2 | 1 |

Note: Metabo Ratio, number of differential metabolites / total differential metabolites in pathway; Rich Factor, number of differential metabolites / total metabolites in pathway background; Fold Enrichment, enrichment magnitude; P.adjust, Benjamini-Hochberg adjusted *p*-value; Class, KEGG pathway classification (M, Metabolism; H, Human Diseases; O, Organismal Systems; G, Genetic Information Processing; E, Environmental Information Processing); Up, number of upregulated metabolites; Down, number of downregulated metabolites.

**Supplementary Table S4. KEGG pathways used for multi-omics intersection analysis**

| **Pathway ID** | **Pathway Name** | **Trans** | ***p*-value (Trans)** | **Metab** | ***p*-value (Metab)** | **Intersection** |
| --- | --- | --- | --- | --- | --- | --- |
| **hsa00260** | **Glycine, serine and threonine metabolism** | **Yes** | **0.0173** | **Yes** | **0.0288** | **Yes** |
| hsa00010 | Glycolysis / Gluconeogenesis | Yes | 0.0281 | No | - | No |
| hsa00020 | Citrate cycle (TCA cycle) | No | - | Yes | 0.0311 | No |
| hsa00220 | Arginine biosynthesis | No | - | Yes | 0.0038 | No |
| hsa00232 | Caffeine metabolism | No | - | Yes | 0.0372 | No |
| hsa00250 | Alanine, aspartate and glutamate metabolism | No | - | Yes | 0.0006 | No |
| hsa00350 | Tyrosine metabolism | No | - | Yes | 0.0229 | No |
| hsa00360 | Phenylalanine metabolism | No | - | Yes | 0.0001 | No |
| hsa00380 | Tryptophan metabolism | No | - | Yes | 0.0280 | No |
| hsa00400 | Phenylalanine, tyrosine and tryptophan biosynthesis | No | - | Yes | 0.0013 | No |
| hsa00470 | D-Amino acid metabolism | No | - | Yes | <0.0001 | No |
| hsa00630 | Glyoxylate and dicarboxylate metabolism | No | - | Yes | 0.0118 | No |
| hsa00910 | Nitrogen metabolism | No | - | Yes | 0.0282 | No |
| hsa00970 | Aminoacyl-tRNA biosynthesis | No | - | Yes | <0.0001 | No |
| hsa01100 | Metabolic pathways | No | - | Yes | 0.0120 | No |
| hsa01210 | 2-Oxocarboxylic acid metabolism | No | - | Yes | 0.0005 | No |
| hsa01230 | Biosynthesis of amino acids | No | - | Yes | <0.0001 | No |
| hsa01232 | Nucleotide metabolism | No | - | Yes | 0.0467 | No |
| hsa01240 | Biosynthesis of cofactors | No | - | Yes | 0.0145 | No |
| hsa02010 | ABC transporters | No | - | Yes | 0.0001 | No |
| hsa04061 | Viral protein interaction with cytokine and cytokine receptor | Yes | 0.0417 | No | - | No |
| hsa04657 | IL-17 signaling pathway | Yes | 0.0396 | No | - | No |
| hsa04668 | TNF signaling pathway | Yes | 0.0495 | No | - | No |
| hsa04724 | Glutamatergic synapse | No | - | Yes | 0.0051 | No |
| hsa04727 | GABAergic synapse | No | - | Yes | 0.0065 | No |
| hsa04742 | Taste transduction | No | - | Yes | 0.0097 | No |
| hsa04933 | AGE-RAGE signaling pathway in diabetic complications | Yes | 0.0421 | No | - | No |
| hsa04964 | Proximal tubule bicarbonate reclamation | No | - | Yes | 0.0015 | No |
| hsa04974 | Protein digestion and absorption | No | - | Yes | <0.0001 | No |
| hsa04976 | Bile secretion | No | - | Yes | 0.0458 | No |
| hsa04978 | Mineral absorption | No | - | Yes | 0.0006 | No |
| hsa05030 | Cocaine addiction | No | - | Yes | 0.0039 | No |
| hsa05031 | Amphetamine addiction | No | - | Yes | 0.0065 | No |
| hsa05034 | Alcoholism | No | - | Yes | 0.0081 | No |
| hsa05142 | Chagas disease | Yes | 0.0429 | No | - | No |
| hsa05143 | African trypanosomiasis | No | - | Yes | 0.0051 | No |
| hsa05144 | Malaria | Yes | 0.0210 | No | - | No |
| hsa05211 | Renal cell carcinoma | No | - | Yes | 0.0414 | No |
| hsa05217 | Basal cell carcinoma | No | - | Yes | 0.0140 | No |
| hsa05221 | Acute myeloid leukemia | Yes | 0.0285 | No | - | No |
| hsa05230 | Central carbon metabolism in cancer | No | - | Yes | <0.0001 | No |
| hsa05231 | Choline metabolism in cancer | No | - | Yes | 0.0098 | No |
| hsa05310 | Asthma | Yes | 0.0135 | No | - | No |
| hsa05323 | Rheumatoid arthritis | Yes | 0.0396 | No | - | No |

Note: Significantly enriched KEGG pathways (*p* < 0.05) from transcriptomics (11 pathways) and metabolomics (34 pathways) were used for intersection analysis. The intersection was determined by matching KEGG pathway IDs between the two omics datasets. One intersection pathway (hsa00260, highlighted) was identified. Trans, Transcriptomics; Metab, Metabolomics; p-value (Trans), p-value from transcriptomics enrichment analysis; *p*-value (Metab), *p*-value from metabolomics enrichment analysis.

# Supplementary Table S5. Differentially Expressed Metabolites in Glycine, Serine and Threonine Metabolism Pathway

| **Metabolite ID** | **Metabolite Name** | **Log₂FC** | **Regulation** |
| --- | --- | --- | --- |
| C00078 | L-Tryptophan | 1.041 | Upregulated |
| C00441 | L-Aspartate-semialdehyde | 0.650 | Upregulated |
| C00430 | 5-Aminolevulinic acid | 0.361 | Upregulated |

Note: Pathway ID: hsa00260, p-value: 0.0288 All 3 metabolites in the glycine, serine and threonine metabolism pathway (hsa00260) showed upregulation in childhood asthma patients compared to healthy controls. P-value for pathway enrichment = 0.0288. Log₂FC values are presented with three decimal places.Total metabolites: 3, Upregulated: 3, Downregulated: 0, Mean Log₂FC: 0.684, Range: 0.361 to 1.041. Log_2_FC: log2-transformed fold change (Asthma vs. Control); Positive values indicate upregulation in asthma patients.

# Supplementary Table S6. Spearman correlation analysis between key metabolites and clinical characteristics

| **Metabolite** | **Clinical parameter** | **r** | ***p*-value** | **95% CI** | **Significance** |
| --- | --- | --- | --- | --- | --- |
| L-Tryptophan | IgE (IU/mL) | 0.298 | 0.110 | −0.068, 0.592 | ns |
|  | Eosinophil (×10⁹/L) | 0.592 | <0.001 | 0.296, 0.788 | *** |
|  | FEV1%pred (%) | −0.119 | 0.531 | −0.467, 0.260 | ns |
|  | FEV1/FVC (%) | −0.199 | 0.292 | −0.527, 0.179 | ns |
| 5-Aminolevulinic acid | IgE (IU/mL) | 0.469 | 0.009 | 0.128, 0.714 | ** |
|  | Eosinophil (×10⁹/L) | 0.506 | 0.004 | 0.174, 0.737 | ** |
|  | FEV1%pred (%) | −0.284 | 0.129 | −0.581, 0.082 | ns |
|  | FEV1/FVC (%) | −0.081 | 0.670 | −0.436, 0.295 | ns |
| L-Aspartate-semialdehyde | IgE (IU/mL) | 0.306 | 0.100 | −0.060, 0.597 | ns |
|  | Eosinophil (×10⁹/L) | 0.358 | 0.052 | −0.003, 0.634 | ns^#^ |
|  | FEV1%pred (%) | −0.234 | 0.214 | −0.549, 0.141 | ns |
|  | FEV1/FVC (%) | −0.044 | 0.818 | −0.400, 0.323 | ns |

Note: Spearman rank correlation coefficients (r) and two-sided p-values are reported. 95% CI was estimated using Fisher z-transformation. ns, not significant; ^#^, marginally significant (p = 0.052). *p < 0.05, **p < 0.01, ***p < 0.001.
